# Keep going in adversity – using a resilience perspective to understand the narratives of long-term social assistance recipients in Sweden

**DOI:** 10.1186/1475-9276-12-8

**Published:** 2013-01-22

**Authors:** Anneli Marttila, Eva Johansson, Margaret Whitehead, Bo Burström

**Affiliations:** 1 Division of Social Medicine, Department of Public Health Sciences, Karolinska Institutet, Norrbacka, 3rd floor, 171 76, Stockholm, Sweden; 2 Division of Global Health/IHCAR, Department of Public Health Sciences, Karolinska Institutet, Nobels väg 9, 171 77 Stockholm, Sweden and Nordic School of Public Health, Box 12133, Gothenburg, 402 42, Sweden; 3 Department of Public Health and Policy, University of Liverpool, Whelan Building, Quad-rangle, Brownlow Hill, Liverpool, L69 3GB, United Kingdom

**Keywords:** Sweden, Social assistance, Narratives, Qualitative, Resilience, Adversity

## Abstract

**Introduction:**

In Sweden, means-tested social assistance serves as a temporary, last resort safety net. However, increasing numbers of people are receiving it for longer periods and about a third has assistance for more than a year. The aim of this study was to explore the ways social assistance recipients manage long lasting adversity and their roles as active, rather than passive, agents in this process, using a resilience perspective.

**Method:**

The study is based on thirteen in-depth interviews with long-term social assistance recipients from diverse areas in Stockholm County. The interviews were guided by narrative inquiry to interpret and construct stories of experiences and are part of a larger qualitative study exploring experiences of living on social assistance in Sweden.

**Results:**

Experiences of cumulative adversity during many years compounded recipients’ difficulties in finding ways out of hardship. They had different strategies to deal with adversities, and many had underlying “core problems”, including mental health problems, which had not been properly resolved. Recipients’ showed resistance in adverse situations. Some made attempts to find ways out of hardship, whereas others struggled mainly to achieve a sense of mastering life. They received important support from individual professionals in different authorities, but mostly the help from the welfare system was fragmented.

**Conclusions:**

Social assistance recipients in this study demonstrated agency in ways of managing long lasting difficulties, sometimes caused by “core problems”, which were often accumulated into complex difficulties. Resilience was about keeping going and resisting these difficulties. To find ways out of social assistance required help from different welfare agencies and professionals and was hindered by the fragmentation of services. This study shows that there is a need for more long-term personalised, comprehensive support, including interventions both to increase individual well-being and self-esteem and to open up opportunities for education and employment. Adequate benefit levels and overall quality of welfare services such as health and social care, day care and schools, are of major importance for those in greatest need.

## Introduction - long-term social assistance recipients at risk of social exclusion

Social exclusion is a major risk for people living long-term in adversity and on welfare benefits. Over many decades, one of the explicit goals of government social policy in Sweden has been social inclusion of citizens at the bottom of the income distribution, and minimizing marginalization [1-3]. Social exclusion is therefore ideologically important, as recognized also by the WHO Commission of Social Determinants of Health [4] and the British Marmot review [5]. Income inequalities have widened in recent times in Sweden [6] resulting in a growing proportion of the population at-risk of poverty.

In Sweden, means-tested social assistance serves as a last resort safety net. In 2010, 4.7% of the population received social assistance at some occasion during the year [7]. Social assistance can be received as a main source of income or as a supplement to other incomes which fall below a specified level. More than one-third (37%) of all recipients aged 18 years and older had social assistance for at least 10 months in a year [7]. Recent Swedish studies [2,8] suggest difficulties in getting off social assistance. Young adults, single mothers and immigrants (especially newly arrived) are common groups among recipients [2,8]. For example 24 per cent of Swedish lone mothers received social assistance on some occasion in 2010 [7].

During the severe economic recession in the 1990s, unemployment increased as did the number of social assistance recipients. Social policies became more restrictive, including cutbacks in public benefits and tighter eligibility criteria to means-tested benefits [9]. The average duration of social assistance recipiency has increased from 4.3 months in 1990 to 6.6 months in 2011 [10]. Hence, the situation of long-term recipients deserves special attention, as their situation may be seen as an indication of the state of welfare in the country.

Levels of social assistance in Sweden as in the other Nordic countries, have been higher than in other welfare state models [3]. Kuivalainen and Nelson [9], however, show the recent development of the erosion the adequacy of benefits in Finland and Sweden. In 2007 Sweden provided less adequate benefits than, for example, the Netherlands and Germany [9]. Kuivalainen and Nelson [9,11] state that current benefit levels do not provide enough money for families to escape poverty in the Nordic countries; in terms of benefit generosity and poverty outcomes Nordic social assistance has moved closer to international patterns.

Studies have shown that both individual and structural factors are associated with receipt of social assistance [2]. Recipients of social assistance in Sweden and Norway, for example, tend to be worse off than non-recipients in terms of educational achievements, financial resources, access to material goods, employment, self-reported health and quality of social relations [1,12]. Exit from long-term social assistance to work is higher for men than for women, and higher for younger people [1,13], but generally low among long-term recipients [1], suggesting that the welfare-to-work programs may be less effective for long-term recipients [14].

Qualitative studies among social assistance recipients in the Nordic welfare contexts have highlighted that shame, feelings of being stigmatised, anxiety, isolation and feelings of insecurity are common [15,16]. Living on social assistance for a long time, in ‘chronic adversity’ [17], also means living with limited material resources, lack of autonomy, and feelings of ‘otherness’ [18], with consequences for health and well-being [15,16]. Some of those already suffering deprivation may accumulate further disadvantages over time, increasing the risk of marginalization and exclusion from mainstream society [18-20].

The concepts of social exclusion and poverty in its widest sense overlap. By poverty some researchers refer to a lack of resources, especially income [21], or to relative aspects [22], whereas in social exclusion the multi-dimensional character is emphasized; components of personal welfare like employment, education, financial resources and health are seen as interrelated and mutually reinforcing [23-25]. Sen [26] discusses poverty in terms of poor living, capability deprivation, and being excluded from social relations which can lead to other deprivations and further limit a person’s life chances. Social exclusion takes place in a specific context; it is relative and dynamic in that not only the present, but also the past and the future are relevant factors in the process of social exclusion [27]. Agency, another aspect of social exclusion [27], is a focus in this article and therefore discussed in more detail. Agency, or the capacity of people to act, has recently gained increasing attention in research related to social exclusion.

### People who are excluded as active agents in their lives

The idea of agency is usually to see individuals as “*autonomous, purposive and creative actors, capable of a degree of choice*” (page 125) [18]. Giddens [28] highlights that agency refers not to the intentions people have in doing things but to their capability of doing. According to Sen [29], functionings concern what a person manages to do and capabilities what a person can do, or can choose in her situation. The focus is on the positive, what kind of life people will be able to live, and how people are able to flourish [18]. Sen [30] states that understanding the agency role is central to recognizing people as responsible persons: *“not only are we well or ill, but also we act or refuse to act, and can choose to act one way rather than another”* (page 190). The central question is the extent to which wider social, economic and political structures enable or constrain the agency of different groups and how their agency can have an impact on the wider structures in society [18].

Lister [18] discusses agency in relation to four mechanisms: ‘getting by’, ‘getting back at’, ‘getting organised’ and ‘getting out’. ‘Getting by’ refers to how people manage and adapt to their circumstances, deal with processes of stigmatization and exclusion and cope with personal trauma and tragedies of past and present [18,31]. ‘Getting back at’ refers to the resistance people in poverty show, for example when dealing with welfare authorities and against negative labelling. ‘Getting organised’ is a collective act, involving the capacity of groups living in poverty for political activism and collective self-help [18]. ‘Getting out’ refers to attempts to move out of poverty and exclusion, which according to Lister [18], can include both individual actions of the poor and the non-poor and economic and social processes and policies. The links between individual agency and the social environmental context has also been highlighted in the resilience literature.

### Agency and resilience

Often, resilience is conceptualised as an individual trait or characteristic: people are either resilient or they are not. There is a small but growing body of literature, however, that conceptualises resilience as a process: “*a dynamic process encompassing positive adaptation within the context of significant adversity*” (page 543) [32]. In this literature, resilience is not a condition of individuals alone but related to vulnerability and protective factors in individuals´ environments [32,33]. Ungar and Liebenberg [34] highlight the importance of both external and internal resources for positive development, which in turn are influenced by culture and context. The focus is on conditions enabling individuals to develop normally despite adverse life conditions [32,35].

The importance of creating settings where individuals have the opportunity for positive development has also been acknowledged [33,36,37]. In the health assets literature [38], several other concepts like sense of coherence [39] and self-efficacy [40] have been discussed, as related to the concept of resilience. Resilience is a relative, culturally and contextually dependent concept; for example, for resilience, important resources like self-esteem and efficacy are more or less valued in different settings [35,36]. In the study reported here we maintain the definition employed in our previous studies, in which resilience is conceptualised as: “*the process of achieving positive and unexpected outcomes in adverse conditions*” (page 238) [41].

People who are poor or socially excluded are often described in research as passive recipients of welfare services [18,42]. Little is known about the ways in which long-term recipients of social assistance are active agents in their lives, or their experiences of the obstacles and possibilities to cease welfare. From a public health perspective it is important to gain deeper knowledge about what in a long-lasting adverse situation makes the situation better or worse for those experiencing it.

### Aim

The aim of this study was to explore and describe the ways long-term recipients of social assistance manage long lasting adversity and act as active, rather than passive, agents in their lives. We were also interested to examine social and health services, and their potential role as either supporting or undermining the well-being of social assistance recipients. We discuss our findings from a resilience perspective.

## Method

This study is based on in-depth interviews guided by narrative inquiry [43-45] to interpret and construct stories of experiences [45]. Narrative methods were judged as suitable as we were interested to explore the experiences of the individuals both in the past, current situation and future prospects, influenced by the surrounding environment in interaction with others. We view the research interview as social encounter, a conversation between two active participants [44,45], led by the researcher around a specific topic, in which meaning is shaped. In this study interviews are understood as narratives, stories told by interviewees about their lives.

### Setting, sample and data collection

Our sample is part of a larger study including 33 interviews, conducted by the first author, with social assistance recipients in Stockholm County (18 men and 15 women) gathered in 2005–2006 through purposeful sampling. Our primary purpose was to explore experiences of living on social assistance in Sweden. The secondary purpose was to gather data in different local environments to contrast interviewees’ experiences. We therefore conducted field work, including interviews, in six socioeconomically diverse areas in Stockholm County (one affluent, one socioeconomically mixed, one outer, and three disadvantaged areas, see Table 1). Fourteen interviewees were recruited through social services, units working with administration of social assistance benefits in each of the study sites, the others through activities to which social assistance recipients were often referred. The sample consisted of persons with different backgrounds, and long experience of social assistance. Most of the interviewees had received social assistance for several years (up to ten years), and many had also received it periodically.

**Table 1:** Characteristics of the interviewees

**Characteristics**	**No of interviewed (13)**
**Gender**	
Female	8 (5 had children)
Male	5 (2 had children)
**Age**	
19–29	5
30–49	7
50+	1
**Ethnic background**	
1. generation immigrant	4
2. generation immigrant	3
Swedish	6
**Education**	
Elementary school	3
Secondary school	6
Dropped out from school	4
**Duration on social assistance**	
1–2 years	4
3–5 years	3
More than 5 years	6
**Study site**	
1 Outer area	4
2 Disadvantaged area, high proportion on social assistance	3
3 Disadvantaged area, less than expected on social assistance	2
4 Affluent area	2
5 Diversity, mixed neighbourhood	1
6 Disadvantaged area, less than expected on social assistance	1

The length of the interviews varied from 40 minutes to two and a half hours. Before the interview took place, the aim of the study was explained and the interviewees were informed of their right to withdraw at any time. An interview guide was used with open-ended questions about the daily lives of the interviewees, their contacts with health and social services, and how they managed their finances. We also asked about how they felt about their health and future. Interviewees reflected on their experiences of living on social assistance and discussed reasons for and consequences of long-term welfare recipiency. Interviews took place at social welfare offices, at local libraries or in activity centres to which interviewees had been referred. The interviews were tape-recorded (with the permission of the interviewees) and transcribed verbatim.

### Analysis

All 33 interviews were analysed with the aim of exploring everyday experiences of living on social assistance [15]. Subsequently we analysed a sub-set of interviews reporting chronic illness in a separate study [46]. The 33 interviews were grouped into three categories based on the main focus of the interviews. We call one category of interviews “unemployment stories sometimes combined with ill health”, a second category “disability stories” and a third “accumulation of adversity”. The content of many of the interviews overlapped categories, in which case allocation to a category was based on the most prominent element of the interview.

In the study presented here we explored the stories in the category “accumulation of adversity”. We organised the transcribed interviews, one by one, into story lines [43], arranging life events chronologically, services and support that the interviewees had received from the society, including turning points in life, in which the told story took a new direction. In this way we could also identify life course stages in the accounts [47].

Subsequently we analysed the interviews thematically based on their content [45], studying the “agency” in the stories (how interviewees managed, reflected and acted in their life situation, and possible ways out of it) and how health and social services buffered or made things worse for the interviewees. Finally, we studied the story lines, life course stages and “agency” together, writing case narratives, retelling the stories [43]. In particular, three dimensions were central in our analysis, in line with the literature on narrative analysis [43,45]: the temporal dimension (past, present and future, the continuity of the story), the interaction between the personal and social dimensions, and that stories occurred in specific places/contexts. Our analysis was case centred, that is, we analysed mainly each case intact instead of analysing segments of data across cases [45]. The analytical strategy and focus, and the categorisation of data were discussed and developed by all the authors.

Our purpose was to study individual cases, in their wider social contexts, seeing public dimensions in private problems [48]. We use the words hardship and adversity as synonyms, and illustrate the findings with some excerpts of the case stories. The names used are pseudonyms. The study underwent ethical review but did not require ethical approval under local guidelines (dnr 04-609/5) (recruitment of the study participants was conducted in a way which did not require identification of interviewees’ personal data such as address or identification number).

## Findings

The sample consisted of thirteen social assistance recipients, both men and women from a range of backgrounds and ages (Table 1). They had received social assistance from between one and ten years, but difficulties with the family, life situation or household finances had lasted for many years, in several cases since their childhood or adolescence.

We present our findings through the main theme; keep going in adversity, and three subthemes: managing accumulating hardship, resisting downward trajectory and ways out of adversity – blind alleys. In interviewees´ accounts, the subthemes occurred as parallel processes and were interwoven with the main theme of keeping going within a context of long term adversity.

### Managing accumulating hardship

**Figure 1:**
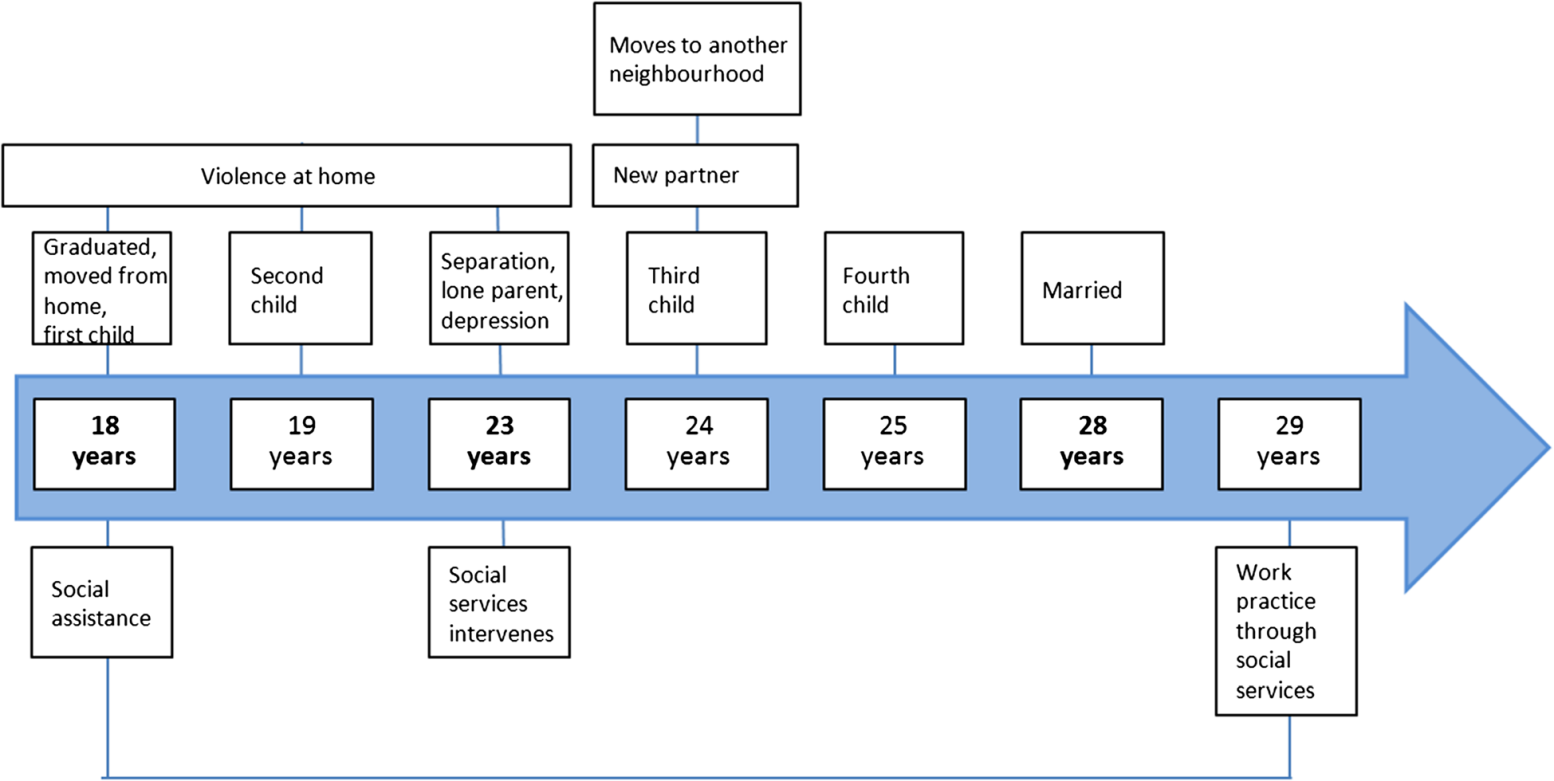
Anna’s story line (major turning points in bold).

The first subtheme concerned recipients’ ways of managing long lasting hardship and strategies to deal with the situation, which we illustrate with an example of one interviewee, Anna and how her life was told (Figure 1). In Anna’s story hardship was discussed in several dimensions and the way out seemed difficult to find. Social and health problems as well as financial difficulties during many years characterized her story. Vulnerability in one aspect seemed to lead and result in vulnerability in other aspects. The help from the society (health and social services) had been fragmented in Anna’s life (as well as several of the other studied stories); only the economic assistance from the society had been continuous.

Anna’s story consisted of four life course stages. Her childhood and adolescence were characterized by growing up in a disadvantaged neighbourhood and with addiction in her family (Figure 1). In the second period, she left her parental home to live with her boyfriend, and had two children. This was a period of surviving in a destructive relationship combined with living in material adversity (disadvantaged neighbourhood, economic strain, behavioral and developmental problems of the children). The third period was a one of depression and building a new family, and the fourth looking forward and bringing up children. Financial strain, insecurity in everyday life and earlier negative experiences resulted in low beliefs concerning the future, but at the same time her children made life meaningful. She told about her “inner strength” for fighting for her family. Below an excerpt from the case narrative illustrates how Anna reflected over the consequences of living long term on social assistance and the present situation.

"Every month was a struggle to make ends meet. Anna told that her children had never been on holiday with her, affording new bikes for the children or letting them participate in a soccer team was also impossible. This and being treated as a citizen with “lower human value” were in Anna´s view the most difficult things about living on social assistance. Presently, she was waiting to start work practice organised through social services, but had little faith in her ability to keep a job as felt she had limited control of her life, “something happened constantly” which made her give up. However, Anna described herself as someone who considers difficult experiences in life as challenges from which you may learn and grow stronger. “They [social workers] ask me how I can deal with all my troubles. They are really positive to me because I am so honest…They see that I am fighting for my family.”"

We found similar phenomena in all stories; vulnerability in several aspects, but also factors reflecting their resilience in the face of adversity. Their strategies to deal with their situations differed.

#### Strategies to survive

Three strategies to survive were identified in interviews: prioritizing family, searching balance in life and reorienting the way of life. In several stories motherhood and family responsibility dominated. In these “family first” stories, such as in Anna’s story, the well-being of children and the family were put first. Interviewees gave examples of how, in a context of limited finances, they put their own needs second to ensure that their children were adequately fed and clothed. When their children fell ill or had troubles, mothers prioritized their children instead of their own participation in the labour market or education.

Some of these mothers described violence in their earlier relationships, and saw it as their own responsibility to sort out the situation. Statements like: “*you have yourself to blame*” were noticeable. Interviewees expressed the view that they had made choices in their life to have their children, had got involved with “wrong kind of partners” and had to take the consequences. Some of the mothers “accepted” that they had to rely on social services for their living. One travelled almost four hours a day for a low paid part-time job and complemented her income with social assistance. She did it for her “*independence*”, and to do her “*duty in society*” although her child had to be in day care ten hours a day and she gained no economic advantage from it.

Some other interviewees, both men and women, discussed their underlying “core problems” such as learning difficulties and failure in school and how that continued to affect them. A few of them also reported mental illness (women) and their insecurity regarding their future. These stories were about “a search for balance in life”. Rebecca for example who suffered from panic attacks since her teenage years talked about her wish to find a balance in life, learn to deal with both negative and positive things in her life.

Several interviewees stated that their most important goal was to reorient their life and cut down on drugs/alcohol (both men and women) or staying away from criminality (men). In these “addiction” or “criminality” stories, feeling well in their “new life”, and to find and keep a flat were crucial. This took time and was not possible in their view without help from welfare agencies. Most of these interviewees had a strong working identity, and long working experience. Getting a job was not perceived as a major obstacle in these stories.

The ways in which the recipients coped with everyday struggle were not always good for their health or could make them feel better in one way and worse in another way. Several interviewees described, for example, how they saved money on food sometimes to afford to “drink a beer or a coffee with friends”, to have a social life as “everybody else”. Smoking was identified by some interviewees as a coping aid, while noting how bad smoking was from an economic point of view in their particular situation.

#### Social belonging in adversity – striving towards a “normal” life

Recipients discussed the ways in which family and friends could help in adversity, not necessarily financially, but they gave clothes, cut hair, provided company on a walk or were just there when needed. Several interviewees had an immigrant background, and not all had relatives in Sweden, which limited their social network. In all stories, the time lived in adversity was long and when difficulties became more intense, interviewees recognized that the number of friendships tended to reduce, and contacts with relatives became less frequent. Some interviewees described that they had either no or very few friends. Those in the process of quitting drug/alcohol abuse reported that social contacts were one of the most difficult parts of the recovery; to create a new way of life cutting off their previous contacts. In some cases, their only contacts were with authorities.

Social belonging to others living in adversity was discussed. As “long-term social assistance recipients” or “persons who had grown up in a disadvantaged neighbourhood” or as “addicts”, the interviewees perceived themselves as “disadvantaged” or “vulnerable”. They had no other choice but to rely on help from the authorities. At the same time, however, interviewees distanced themselves from the “most disadvantaged”, and did not want to be described as one of “them”. For example several interviewees described segregation in their neighbourhood and how “*totally different people moved in compared to earlier*”. A desire to lead “a normal” life characterized the stories.

### Resisting downward trajectory

The second sub-theme concerned resistance, persisting, in adverse situations, where recipients´ well-being was threatened, the situation was out of their control, and they sought help from outside. Resistance was also about interviewees´ convictions about what would be the best to do in their situation. Interviewees discussed the importance of sustained contact with professionals in health and social services in order to provide an opportunity to build trust and confidence and in a long run open up possibilities for them to move in an upwards trajectory.

#### Critical moments in life- seeking help from authorities

In each story we identified critical moments, times in life when interviewees perceived they could not manage their situation by themselves but contacted different authorities such as the police, social services or health care. Interviewees expressed an appreciation for receiving help quickly in these situations, when they stood at a crossroad; their life could take different directions depending on the support and help they received. In these situations they were motivated to accept help. Sometimes the service providers succeeded in offering help that matched their needs. Sonja recounted how the police helped her to turn her life around in a very difficult situation. She talked about how help she received from different authorities one after another helped her forward, strengthening her own resilience to change her life.

"Sonja, a 44 years old woman with immigrant background, had been drinking “too much” for many years, but especially after the death of her husband. “Without children and alone, I started to hang out with my friends and drank more and more.” She lost her flat and moved to a new partner, who soon became violent. Sonja said: “I don´t understand why I stayed with him. I have always thought that I would never be with someone who is violent. But I was so down…” Sonja called the police once when her partner got angry and violent. They arrested him and came to meet her the day after saying they would support her if she decided to report her partner, who had been violent also in his earlier relationships. “They asked me if I wanted to live. To go on in that relationship would not lead to something better.” After that Sonja described “everything has gone well”. Support from different authorities had carried her forward. She had succeeded to quit drinking and managed with the help of social services to find a new flat. She had been open with her problems, willing to change her life, which she perceived had helped her in her contacts with the authorities."

To find someone who “really tried to help” was what made the difference. Even if it was not always possible to get help and support right away, it raised the opportunity of longer term sustained contact. Samir told about his experiences when he left prison aged 18, and the support he got from a social worker, who believed in his willingness and ability to change his life.

"Samir had been in contact with social services since he was 7 years because of criminality, having grown up in a disadvantaged neighbourhood and hanging out with “criminals”. At 18 he was sent to prison which he perceived was “the best thing that happened to him”, he had time to reflect over his life, and decided to change his way of life. He called social services from the prison to help him find somewhere to live. They could not and he became homeless, which was “an awful period” moving between his friends from day to day. However, a social worker tried to help him and kept contact with him during the six months when he was homeless. With her help he found a flat, something of his “own”. Samir described the social worker as “someone worth gold”, a person who he could trust. He had not felt trust in the social workers he had been in contact with earlier."

Interviewees discussed their interaction with welfare services, which often was fragmented, and it was often difficult to receive on-going and sustained support, other than financial, especially with some of their underlying problems. Some interviewees perceived they had never been recognised as “real individuals”, but rather as one among many. Susanna for example recounted her experiences of living with back pain over ten years, and how different doctors without seeing her prescribed medication instead of investigating why she had pain. She repeatedly contacted both social and health services to get help because she “did not feel well”. She perceived her life as a “disaster”, being almost dependent on pain killers, which increasingly took over her life; making it more difficult to function in everyday life. She wanted and sought help, but from Susanna´s perspective, she did not receive the help she needed.

#### Goals in line or in collision with authorities and services

In many cases participating in activities, to which social services referred the interviewees, was perceived as “not leading anywhere”. Rebecca for example had been in work practice over six months, with hope of getting employment. She was disappointed when she realised that would not happen (she had been informed that she would not be offered an employment). Work experience was, however, generally described positively, as it gave interviewees the opportunity to build their experience in the labour market and helped to clarify their interest in a specific field of work.

Some interviewees described other specific interventions such as therapy for panic attacks or discussion groups for addicted women, as helpful for improving well-being and functioning in everyday life. Very few interviewees, however, had a long-term plan for addressing their “core problems”, such as mental ill health or learning difficulties. In interviewees´ stories different public agencies often acted separately instead of jointly. In several cases interviewees described how they tried to handle the complexity of their multiple contacts with these different actors.

### Ways out of adversity – blind alleys

The third sub-theme - ways out of adversity, blind alleys - describes interviewees’ attempts to find ways out of hardship and overcome obstacles along the way. None of the interviewees had a clear idea about how to become economically independent. Some talked about finding a job while others talked about obtaining an education in the future. Some interviewees were in the process of improving their well-being following addiction problems or problems with mental ill-health. Many of the interviewees described ways they had attempted to overcome their challenges, but they also reflected that they often had little success.

#### Attempts with jobs and education

Interviewees wished to live an independent life in the future but had difficulty seeing how that could be realised, given their obstacles. They had lived on social assistance or been outside the labour market for many years, had limited education, and in several cases inadequate work experience and limited opportunities to find a job. Interviewees also found limited opportunities for support to enter the labour market in an incremental way, to enable them to balance work with their households’ commitments taking into account their health status.

Several interviewees (women) with children explained that they wanted to find a job, but in the future they dreamed of educating themselves. Josefin for example wanted to study midwifery. Anna saw education as a possibility when her children were older. Education was not an option for everybody. In particular, the interviewees who had described learning difficulties recounted their negative experiences. Susanne for example had made “five or six” attempts to finish secondary school, without any specific help. Every time she had failed.

"“I do like to study but I cannot follow the teaching. I don’t hear that well when all are talking at the same time. I look like a question mark. I don’t understand the books…I don’t know what I have read, I forget…I give up after a week.”"

#### Better functioning/mastering

For several interviewees better well-being and mastering was necessary before they could try to find solutions to their money problems. Rebecca was one of them. For her it was most important to “become stronger” and believe in her capacity to manage challenges. Mental ill health colored Rebecca’s story, which also illustrated difficulties for a young woman with learning difficulties to find her way in life. Rebecca found joy in being with her boyfriend she recently met and taking care of her dogs.

"Rebecca was a 24 year old single woman with Swedish background. She described her teenage years as “bad years” in her life. She did “not feel well, did not bother what happened”, and drank alcohol almost daily (aged 13 to 17). She had difficulty to understand and follow the teaching in school; especially maths, and got no help with her learning difficulties. She described that during her teenage years her problems with panic attacks had got worse. Only when she was 17 years old did the school teachers take action on her behavior. Her parents were shocked. She dropped out of school and got treatment for her panic attacks."

"She left home at the age of 18 when she succeeded to find a flat. “I decided that I have to take responsibility”. Since then she had received social assistance. Rebecca perceived that she received good help for her problems with panic attacks. Social services decided to step by step increase their demands concerning her participation in the labour market activities that they offered her. For her it was most important to become stronger, overcome her sense of vulnerability. She found peace and joy in her dogs, walking them and taking care of them. Her dream was to move to the countryside with her boyfriend, to get “a new start” in her life."

Like Rebecca, all interviewees identified “assets” in their lives, such as children (Anna), inner strength (Anna and Sonja), or positive experiences from professionals (Sonja and Samir), which gave them strength, provided meaning in their life, and helped them to keep going and function despite the adversity they experienced. Some interviewees had more faith in their capacity than others, believing that “*it is somehow going to be all right*”, seeing the world as manageable and comprehensible despite obstacles. Others had previous negative experiences and needed more support in strengthening their self-esteem and belief in their own capacity, that a positive outcome is possible.

## Discussion

Long-term welfare clients are often discussed as passive recipients of services [18,42]. We studied the interviewees as active agents in their own lives [18,30], and interpret our findings from a resilience perspective.

### Multi-layered vulnerability

All studied narratives were handled as unique stories of individual life histories. Social assistance recipients in Sweden are a heterogeneous group, with different backgrounds and challenges over the life course, which has been discussed elsewhere in the literature [1,2,14,25]. This also characterized the interviewees in our sample. Common to all interviewees was that they had struggled with different kinds of problems for many years and were in need of support from the society.

In many stories we also found one or more “core problems” which had not been properly addressed and resolved, and thus contributed to a downward spiral and accumulating adversity over the life course [19,20], also reported in the social exclusion literature [19,25,49]. Such core problems in our study included problems in the childhood family, learning difficulties, mental and other health problems, and domestic violence. These were further compounded by a range of other obstacles, for example poverty, alcohol/drug abuse, living in a disadvantaged neighbourhood, and social isolation, into complex difficulties in everyday life. Vulnerability in one aspect tended to lead to vulnerability in other aspects; interviewees had limited opportunities to make choices in their lives.

### Ways of managing adversity – agency and resilience

In our study, agency concerned interviewees’ managing of long lasting difficulties, resisting downward trajectory and search for finding ways out of financial and social difficulties. Resilience in the studied interviews was not about something ‘extraordinary’ among special individuals, but as Masten [50] and Lindström and Eriksson [36] found, rather it involved ordinary processes to overcome obstacles in hardship. In this study, examples of agency included nurturing children to grow and develop normally despite adverse conditions and not giving up the hope of educating themselves despite learning difficulties were such examples. We found a similar phenomenon when we studied British families living in poverty [41]. Resilience was about resistance, not giving up but trying to find solutions and escape routes out of poverty and adverse situations, especially in critical moments in life, when interviewees were motivated and open to changes in their lives, and accepted external help.

We found evidence of ‘assets’ that interviewees possessed, which strengthened them in adversity and gave meaning in their lives, in line with the assets-based model of health development promoted by the World Health Organisation [38]. Antonovsky’s concept of ‘sense of coherence’ [39] and Bandura’s notion of self-efficacy [40] were of relevance in understanding what factors helped the interviewees cope and achieve positive outcomes in adversity. However, positive outcomes did not depend only on the individual, but as Bartley et al. [33] acknowledge: “*the more time the individual has spent in a capability-producing environment, the greater the resilience they are able to carry forward to meet the next challenge they may face*” (page 104).

In interviewees’ stories, children were perceived as a strengthening factor in their lives. In our study, women were the primary caregivers for their children. For these women, their children buffered their resilience and well-being; however, their caring responsibilities also limited the time they could work. Graham [51] and Lister [18] discuss the hidden female poverty, how women on low income in the UK studies sacrificed their own needs for other family members. Our Swedish findings indicate similar sacrifices. Several women talked about their strategies to guarantee a certain level of well-being for their children, putting their own needs second, and were partly blaming themselves for their difficulties, but also attributing part of their difficulties to factors in their social context and social structures beyond their control, such as addiction or violence in their family or discrimination in the labour market.

### How can services enhance resilience?

A review of qualitative studies conducted in the UK of experiences of informal and formal support among low-income parents concluded that the perception of being supported is as important as the actual support received [52]. In our study those interviewees who perceived that they had obtained help from the welfare services and had met a professional they trusted, also reflected over other factors which strengthened their resilience and helped them to function and feel better in their situation (an upward moving spiral as conceived by Canvin et al. 2009 [41]).

However, motivating or strengthening the self-esteem and self-efficacy of clients was not enough in situations where support in entering the labour market and education was also needed. Learning difficulties, mental ill-health, addiction problems and in several cases domestic violence over many years, added to the difficulties and required more comprehensive, individualised long-term support. In the narratives in our study, the support from the welfare system, including health and social care, seemed to be fragmented. In most cases only the economic assistance had been long-term.

Resilience in the interviewees’ stories was about resistance, not giving up but trying to find escape routes out of poverty and adverse situations, especially in critical moments in life, when interviewees were motivated and open to changes in their lives. That is an important aspect to notice in both health and social services in contact with individuals and families living in adversity. Social and health services have an important role to support social assistance recipients towards inclusion, by recognising them as active agents with their experiences in the past and desires for their future, and the need to increase recipients possibilities to participate in decisions affecting their lives [15].

### Limitations of the study

This study concerns stories of thirteen individuals in the Swedish system and does not tell us about how common mental ill-health, learning difficulties or violence at home is among long-term social assistance recipients in general. More knowledge is needed about these aspects in order to improve and develop adequate support and early interventions for those in need. In this study gender and ethnicity aspects were only briefly mentioned. However, both gender and ethnicity are highly relevant aspects to explore more in depth and to take into account in developing interventions to social assistance recipients.

## Conclusion

Agency, or the capacity of people to act, was evident in the ways that social assistance recipients devised to manage long lasting difficulties, sometimes caused by “core problems”, which were often accumulated into complex difficulties. The process of resilience was also evident in the examples of interviewees keeping going and resisting these difficulties. Finding ways out of social assistance required help from the society and professionals and was hindered by the fragmentation of services. Tackling reported “core problems”, such as mental ill-health and learning difficulties, would require more long-term personalised and comprehensive support than interviewees had received. This study indicates that there is a need for such interventions both to increase the individuals’ well-being and self-esteem, and to open up opportunities for education and employment. Adequate benefit levels and overall quality of welfare services such as health and social care are of major importance for those in greatest need and their children, in order to support resilience and minimise social exclusion.

## Competing interests

The funding organisations had no role in the study design, data collection, analysis, interpretation or writing this article. The authors declare that they have no competing interests.

## Authors’ contributions

AM participated in the design of the study, carried out the interviews, analysis of the data and drafted the manuscript. EJ participated in the analysis of the data and preparing the manuscript. MW and BB participated in the design of the study, in the analysis and preparing the manuscript. All authors have read, revised and approved the final version.
